# Recent trends in psychotropic medication use in children and adolescents in Ireland

**DOI:** 10.1007/s00787-024-02530-7

**Published:** 2024-08-08

**Authors:** Rebecca Parkin, Kathleen Bennett, Fiona Mc Nicholas, John C. Hayden

**Affiliations:** 1 School of Pharmacy and Biomolecular Sciences, RCSI University of Medicine and Health Sciences, Dublin, Ireland; 2 Data Science Centre, School of Population Health, RCSI University of Medicine and Health Sciences, Dublin, Ireland; 3 Department of Child and Adolescent Psychiatry, School of Medicine and Medical Science, University College Dublin, Dublin, Ireland; 4 Lucena Clinic, Rathgar, Dublin, Ireland; 5 Children’s Health Ireland, Crumlin, Dublin, Ireland

**Keywords:** Psychotropic medication use, Trends, Dispensing, Children, Adolescents, Ireland

## Abstract

In response to concerns regarding overprescribing of psychotropic medication in children/adolescents, this study examined trends in psychotropic medication use in Ireland by age group and gender. A retrospective, repeated, cross-sectional study of the Irish pharmacy claims database was conducted. Yearly prevalence of children/adolescents receiving dispensed psychotropic medications was analysed from January 2017 to December 2021 and compared across years, age groups (5–15 years, and stratified as 5–11 and 12–15 years) and gender. Yearly prevalence was defined as the mean number of patients in receipt of medication per month per 1000 eligible population during a given calendar year. Negative binomial regression was used to examine the association of year, age group and gender on prevalence. Prevalence ratios (PRs) per year (average change in prevalence between each year) were presented with 95% confidence intervals (CIs). The prevalence of included psychotropic medications dispensed in the 5–15 years group increased from 6.41 (95% CI: 6.22, 6.59) in 2017 to 8.46 (95% CI: 8.26, 8.68) in 2021 per 1000 eligible population (32% increase). The PR per year (adjusting for age category and gender) was 1.07 (95% CI: 1.035, 1.107; *p* < 0.001). An increasing trend over time was also observed for all individual drug classes. These findings suggest increased use of psychotropic medication in children/adolescents from 2017 to 2021. However, despite increased prevalence over time, comparison with the literature shows that psychotropic medication use in Ireland remains lower than international comparators.

**Supplementary Information:**

The online version contains supplementary material available at 10.1007/s00787-024-02530-7.

## Introduction

The growing prevalence of neuropsychiatric disorders in children/adolescents is a leading concern, with an estimated worldwide-pooled prevalence of 13.4% [1]. This increase in diagnosis of mental disorders in children/adolescents has been paralleled by a rise in prescribing of most classes of psychotropic medications in this population internationally [2–4]. Rates of prescribing and safety issues have recently come under specific scrutiny in Ireland. In January 2023, an interim report published by the Mental Health Commission in Ireland reported significant deficits in child and adolescent mental health services (CAMHS), including long waiting lists, staffing problems, children “lost” in the system (due to insufficient follow-up) and restricted emergency CAMHS out-of-hours services [5]. These findings arose one year after a review was undertaken of a regional CAMHS Ireland centre (South Kerry, Ireland) between July 2016 and April 2021, leading to publication of the Maskey Report. The Maskey Report determined that care received by 240 patients failed to meet required standards [6]. Unreliable diagnoses, inappropriate prescribing and poor monitoring of adverse effects were reported, along with raised concerns of similar risks in other parts of Ireland. Concerns of overprescribing have also featured in widespread media commentary [7, 8].

In the wake of both reports and recent media concerns regarding overprescribing, it is considered timely to re-examine trends in psychotropic medication use in children/adolescents in Ireland. Although earlier Irish studies have reported on CAMHS psychotropic medication use, these studies have been limited to specific classes of medications or subgroups of children. We aim to provide an updated and contemporaneous estimate of psychotropic medication use in children/adolescents in Ireland and compare with international trends.

## Methods

### Study population and study design

A retrospective, repeated, cross-sectional study was conducted based on pharmacy claims data in the community setting in Ireland. Data were obtained from the Irish pharmacy claims database, the Health Service Executive-Primary Care Reimbursement Services (HSE-PCRS). The PCRS is part of the HSE and is responsible for making payments to healthcare professionals, such as community pharmacists, for the free or reduced cost services/medication they provide to the public.

Under the HSE-PCRS system, three principal community drug schemes exist [9]: (i) General Medical Services (GMS) scheme, (ii) Long-Term Illness (LTI) scheme and (iii) Drug Payment (DP) scheme. In order for a medication cost to be covered on one of the community drug schemes, the medication must appear on the HSE list of reimbursable items.

The GMS scheme entitles individuals to primary care services and hospital services free at the point of access, except for prescription medications, which are subject to a flat co-payment (as of August 2023, €1.50 per item, capped at €15 per month per household). Qualification for the GMS scheme is based on income-related means-testing [9]. The LTI scheme is awarded, regardless of income, to individuals with one of 16 chronic illnesses, e.g. diabetes mellitus, epilepsy or mental health conditions (under 16 years). Individuals in receipt of care on this scheme are provided with prescription medicines without co-payment, but general practitioner (GP) consultation fees are not included. The remainder of the population can receive government-subsidised prescription medications through the DP scheme. Under the DP scheme, an individual or family is responsible for paying up to €80 per month (as of August 2023) for their medications [9].

The HSE-PCRS database contains basic demographic information and details of dispensed medications (coded using the World Health Organisation [WHO] Anatomical Therapeutic Chemical [ATC] classification) for each individual on any of the three PCRS schemes. No information on diagnosis or disease condition is available. Data are freely available with the necessary confidentiality agreements. Permission was given by the data controller to use the dataset if anonymised and analysed at group level. Hence, it was unnecessary to seek specific ethical approval for this study. Private prescription data were not included in the analysis as this is not centrally collected by the health system. However, given that medications for mental illness in patients under 16 years are universally reimbursable to community pharmacies through the community drug schemes, such prescriptions should be minimal. Data for four of the most commonly used classes of psychotropic medications (ADHD medications, antidepressants, antipsychotics and hypnotics/sedatives [including benzodiazepines and Z-drugs]) were extracted from the HSE-PCRS database (refer to Appendix 1 for complete list of psychotropic medications included).

Patients or the public were not involved in the design of the study. There were no adjustments made to account for any potential bias in the selection of participants for the study.

### Data analysis

All available data were used and formal cohort size calculations were not included. Yearly medication prevalence, defined as the mean number of patients in receipt of each medication per month per 1000 eligible population (entire 5–15, 5–11 or 12–15 years population) during a given calendar year, was calculated for children/adolescents aged 5–15 years (including stratified age groups 5–11 and 12–15 years) from January 2017 to December 2021. To calculate the yearly prevalence for a specific calendar year, an average of the 12 monthly prevalences for that year was taken. As discontinuation rates of psychotropic medications in youth are high, it was felt that an average number of patients dispensed medication per month (averaged over a year) would better reflect actual medication use compared to a prevalence of those who received any prescription supply during that year. Of note, this did not exclude patients who were not 12-month medication users, but rather it provided an estimate of both 12-month users and partial year users per month in each study year. The alternative approach of counting partial year users and 12-month users equally would overestimate the actual number of youth on medication each month and similarly, would double count those who switched between medications in that year. Associated 95% confidence intervals (CIs) were also calculated. Medication prevalences were then compared across years, age (5–15 years overall, 5–11 and 12–15 years) and gender. Population data (most recent) for the entire 5–15, 5–11 and 12–15 years population were obtained from the Central Statistics Office, Census 2016.

A negative binomial regression model was used to determine trends over time in prevalence. The log of the eligible population was used as the offset term, and year, age group (5–11 years [reference], 12–15 years) and gender (female [reference], male) were included as fixed effects in the model. Interactions between these variables were tested for each of the models. Results are presented as prevalence ratios (PRs) for each variable with 95% CIs (Tables 1, 2, 3 and 4). The PR indicates the average change in prevalence between each year.

Data analysis was performed using Stata version 17 (StataCorp, College Station, Tx, USA).

### International comparison studies

For comparison purposes, international psychotropic medication prevalence data were retrieved from a search of published literature from 2020 to 2023. International studies examining the prevalence of psychotropic medications were identified. Articles were included if they examined trends in use of any or all of the psychotropic medications listed above.

## Results

### Population cohort

During the study period, the eligible population, as identified from the Central Statistics Office, Census 2016, was children/adolescents in Ireland aged 5–15 years (eligible population = 736,680), including 5–11 years (eligible population = 484,368) and 12–15 years (eligible population = 252,312) groups. For each age group, 49% of children/adolescents were male and 51% were female.

### Trends in psychotropic medication use over time for all selected psychotropic medications

**Fig. 1:**
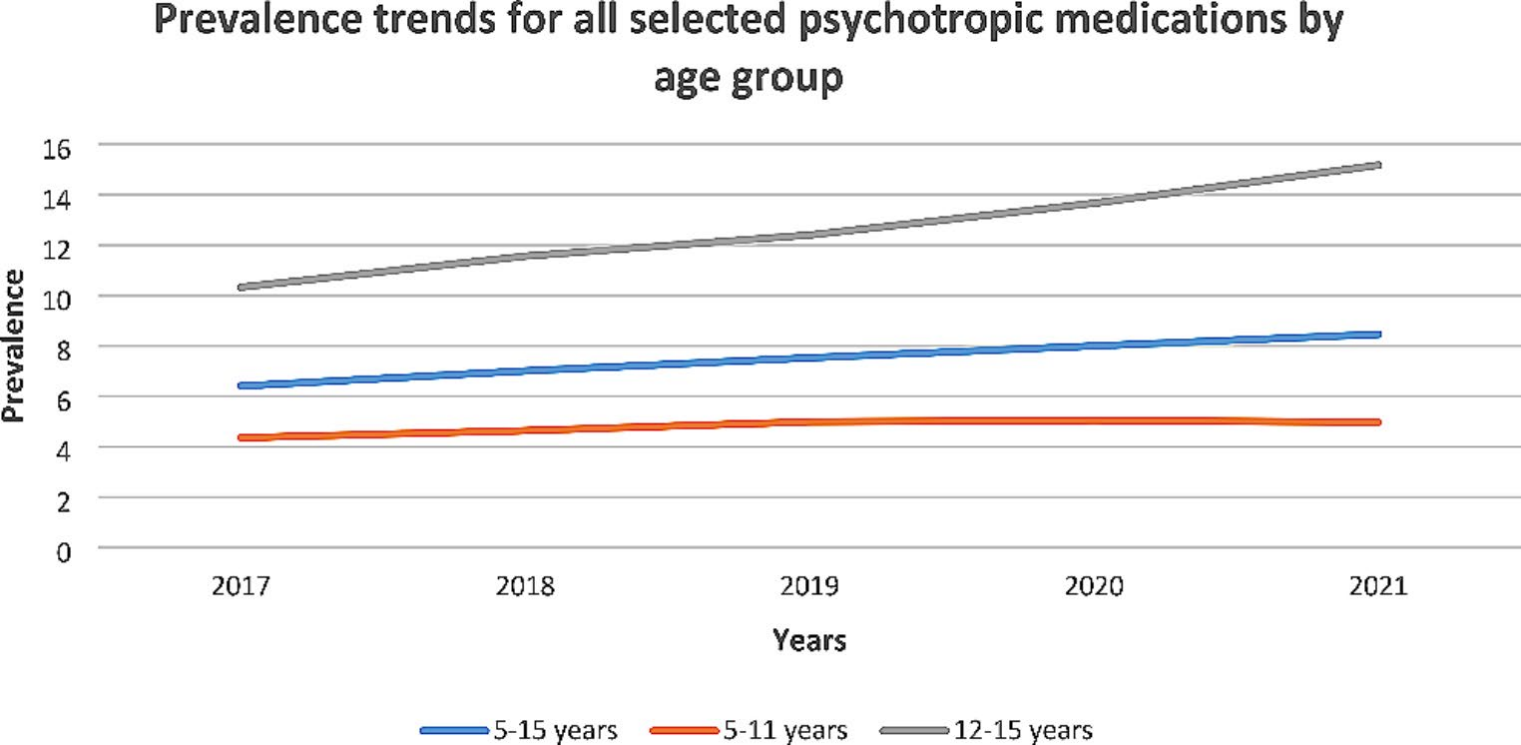
Prevalence trends for all selected psychotropic medications by age group

There was an increasing trend in prevalence for all selected psychotropic medications i.e. all drugs selected for the study, in children/adolescents aged 5–15 years, and also in the 5–11 and 12–15 years age groups (Table 1; Fig. 1). The prevalence of all selected psychotropic medications in the overall 5–15 years group increased from 6.41 (95% CI: 6.22, 6.59) in 2017 to 8.46 (95% CI: 8.26, 8.68) in 2021 per 1000 eligible population (32% increase). The PR (average change in prevalence of use between each year), adjusting for age category and gender, was 1.070 (95% CI: 1.035, 1.107; *p* < 0.001). The prevalence of all selected psychotropic medications in the 5–11 years group increased from 4.36 (95% CI: 4.18, 4.55) to 4.97 (95% CI: 4.77, 5.17) per 1000 eligible population (14% increase). The prevalence of all selected psychotropic medications in the 12–15 years group increased from 10.33 (95% CI: 9.94, 10.74) to 15.17 (95% CI: 14.70, 15.66) per 1000 eligible population (47% increase).

**Table 1:** Trends in prevalence for all selected psychotropic medications by age group, % increase 2017–2021 and prevalence ratio (PR)

Age (years)	Year					% increase 2017–2021	PR^b^ (5–15 years)
	2017	2018	2019	2020	2021		
	Prevalence^a^						
**5–15 (overall)**	6.41 (95% CI: 6.22, 6.59)	7.01 (95% CI: 6.82, 7.20)	7.53 (95% CI: 7.33, 7.73)	8.00 (95% CI: 7.80, 8.21)	8.46 (95% CI: 8.26, 8.68)	32	1.070 (95%CI: 1.035, 1.107; *p* < 0.001)
- **5–11**	4.36 (95% CI: 4.18, 4.55)	4.64 (95% CI: 4.45, 4.83)	4.99 (95% CI: 4.79, 5.19)	5.05 (95% CI: 4.86, 5.26)	4.97 (95% CI: 4.77, 5.17)	14	
- **12–15**	10.33 (95% CI: 9.94, 10.74)	11.56 (95% CI: 11.10, 12.00)	12.40 (95% CI: 12.00, 12.80)	13.66 (95% CI: 13.20, 14.10)	15.17 (95% CI: 14.70, 15.66)	47	

^a^Prevalence = mean number of patients in receipt of each medication per month per 1000 eligible population (entire 5–15, 5–11 or 12–15 years population) during a given calendar year

^b^PR = average year to year ratio in prevalence (average change in prevalence between each year)

### Trends in psychotropic medication use over time for individual selected drug classes

**Fig. 2:**
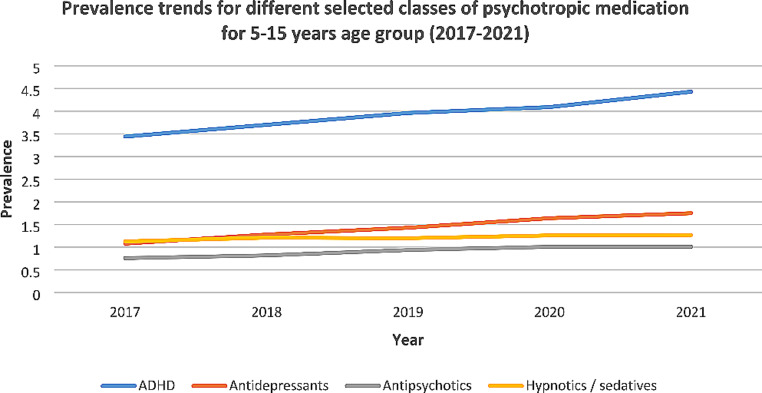
Prevalence trends for different selected classes of psychotropic medication (5–15 years)

An increasing trend in prevalence was observed for all individual selected drug classes within each age group (Table 2; Fig. 2). In the overall 5–15 years group, the prevalence for ADHD medication increased from 3.44 (95% CI: 3.31, 3.58) in 2017 to 4.43 (95% CI 4.28, 4.59) in 2021 per 1000 eligible population (29% increase); PR = 1.064 (95%CI: 1.043, 1.085; *p* < 0.001). For antidepressants, the prevalence increased from 1.08 (95% CI: 1.00, 1.16) to 1.75 (95% CI: 1.65, 1.84) per 1000 eligible population (62% increase); PR = 1.114 (95% CI: 1.046, 1.187; *p* = 0.001). For antipsychotics, the prevalence increased from 0.76 (95% CI: 0.7 to 0.82) to 1.01 (95% CI: 0.94, 1.09) per 1000 eligible population (34% increase); PR = 1.095 (95% CI: 1.033, 1.161; *p* = 0.002). Finally, for hypnotics/sedatives, the prevalence increased from 1.13 (95% CI: 1.05, 1.21) to 1.27 (95% CI: 1.19, 1.35) per 1000 eligible population (13% increase); PR = 1.028 (95% CI: 1.007, 1.050; *p* = 0.008). Whilst ADHD medication remained the most commonly prescribed class of drugs, rates of increase were more prominent in antidepressant prescriptions.

**Table 2:** Trends in prevalence for psychotropic medications in children and adolescents by selected drug class and age group, % increase 2017–2021 and prevalence ratio (PR)

Age (years)	Year					% increase 2017–2021	PR^b^ (5–15 years)
Drug class	2017	2018	2019	2020	2021		
ADHD medication	Prevalence^a^						
**5–15**	3.44 (95% CI: 3.31, 3.58)	3.70 (95% CI: 3.56, 3.84)	3.96 (95% CI: 3.82, 4.11 )	4.09 (95% CI: 3.95, 4.24)	4.43 (95% CI: 4.28, 4.59)	29	1.064 (95%CI: 1.043,1.085; *p* < 0.001)
- **5–11**	2.60 (95% CI: 2.46, 2.75)	2.76 (95% CI: 2.62, 2.91)	2.93 (95% CI: 2.78, 3.09)	2.88 (95% CI: 2.73, 3.04)	2.88 (95% CI: 2.73, 3.03)	11	
- **12–15**	5.06 (95% CI: 4.79, 5.35)	5.50 (95% CI: 5.21, 5.79)	5.93 (95% CI: 5.64, 6.24)	6.42 (95% CI: 6.11, 6.74)	7.42 (95% CI: 7.09, 7.76)	47	
**Antidepressants**							
**5–15**	1.08 (95% CI: 1.01, 1.16)	1.28 (95% CI: 1.20, 1.36)	1.43 (95% CI: 1.34, 1.51)	1.64 (95% CI: 1.55, 1.73)	1.75 (95% CI: 1.65, 1.85)	62	1.114 (95%CI: 1.046,1.187; *p* = 0.001)
- **5–11**	0.33 (95% CI: 0.28, 0.39)	0.35 (95% CI: 0.30, 0.41)	0.40 (95% CI: 0.34, 0.46)	0.45 (95% CI: 0.39, 0.51)	0.44 (95% CI: 0.38, 0.51)	34	
- **12–15**	2.52 (95% CI: 2.33, 2.72)	3.05 (95% CI: 2.84, 3.28)	3.40 (95% CI: 3.18, 3.64)	3.92 (95% CI: 3.68, 4.18)	4.25 (95% CI: 4.00, 4.52)	69	
**Antipsychotics**							
**5–15**	0.76 (95% CI: 0.70, 0.82)	0.82 (95% CI: 0.76, 0.89)	0.94 (95% CI: 0.87, 1.01)	1.01 (95% CI: 0.94, 1.09)	1.01 (95% CI: 0.94, 1.09)	34	1.095 (95%CI: 1.033,1.161; *p* = 0.002)
- **5–11**	0.38 (95% CI: 0.32, 0.43)	0.41 (95% CI: 0.35, 0.47)	0.51 (95% CI: 0.45, 0.58)	0.54 (95% CI: 0.48, 0.61)	0.51 (95% CI: 0.45, 0.57)	37	
- **12–15**	1.48 (95% CI: 1.33, 1.64)	1.61 (95% CI: 1.46, 1.78)	1.76 (95% CI: 1.60, 1.93)	1.91 (95% CI: 1.74, 2.09)	1.98 (95% CI: 1.81, 2.16)	34	
**Hypnotics / sedatives**							
**5–15**	1.13 (95% CI: 1.05, 1.21)	1.21 (95% CI: 1.13, 1.29)	1.20 (95% CI: 1.12, 1.28)	1.26 (95% CI: 1.18, 1.35)	1.27 (95% CI: 1.19, 1.35)	13	1.028 (95%CI: 1.007,1.050; *p* = 0.008)
- **5–11**	1.05 (95% CI: 0.97, 1.15)	1.11 (95% CI: 1.02, 1.21)	1.14 (95% CI: 1.05, 1.24)	1.18 (95% CI: 1.09, 1.29)	1.14 (95% CI: 1.05, 1.24)	9	
- **12–15**	1.27 (95% CI: 1.13, 1.41)	1.39 (95% CI: 1.25, 1.55)	1.31 (95% CI: 1.17, 1.46)	1.41 (95% CI: 1.27, 1.57)	1.52 (95% CI: 1.37, 1.68)	20	

^a^Prevalence = as defined in Table 1

^b^PR = as defined in Table 1

### Gender differences in psychotropic medication use

Prevalences for all selected psychotropic medications and selected individual drug classes, for the overall 5–15 years group, were also analysed by gender (Table 3).

In 2021, the prevalence for all selected psychotropic medications was 11.80 (95% CI: 11.45, 12.16) for males and 5.27 (95%CI: 5.04, 5.51) for females. For the five year study period (2017–2021), the PR for males versus females was 2.477 (95% CI: 2.252, 2.726; (*p* < 0.001). However there was a greater overall percentage increase in prevalence from 2017 to 2021 for females compared to males (41% versus 28%).

**Table 3:** Trends in prevalence for all selected psychotropic medications (5–15 years) by gender, overall % increase and prevalence ratio (PR)

Gender	Year					% increase 2017–2021	PR^b^ (M v F)
	2017	2018	2019	2020	2021		
	Prevalence^a^						
**Male**	9.20 (95%CI: 8.89, 9.52)	10.08 (95%CI: 9.75, 10.41)	10.82 (95%CI: 10.48, 11.16)	11.35 (95%CI: 11.0, 11.7)	11.80 (95%CI: 11.45, 12.16)	28	2.477 (95% CI: 2.252, 2.726; (*p* < 0.001)
**Female**	3.73 (95%CI: 3.53, 3.93)	4.07 (95%CI: 3.86, 4.28)	4.38 (95%CI: 4.17, 4.59)	4.80 (95%CI: 4.58, 5.02)	5.27 (95%CI: 5.04, 5.51)	41	

^a^Prevalence = as defined in Table 1

^b^PR = as defined in Table 1

Similarly, for the individual drug classes (5–15 years group), variations in prevalence according to gender were observed (Table 4).

In 2021, for ADHD medication, the prevalence was 7.31 (95%CI: 7.03, 7.59) for males and 1.68 (95%CI: 1.55, 1.81) for females. For the study period (2017–2021), the PR was 4.497 (95% CI: 4.241, 4.769; *p* < 0.001) in males compared to females. However, there was a greater overall percentage increase in prevalence from 2017 to 2021 for females compared to males (35% versus 27%).

In 2021, for antidepressants, the prevalence was 1.65 (95%CI: 1.52, 1.79) for males and 1.84 (95%CI: 1.70, 1.98) for females. For the study period, the PR was 1.276 (95% CI: 1.063, 1.531; *p* = 0.009) in males compared to females. However, there was a greater overall percentage increase in prevalence from 2017 to 2021 for females compared to males (77% versus 47%).

In 2021, for antipsychotics, the prevalence was 1.42 (95%CI: 1.30, 1.55) for males and 0.63 (95%CI: 0.55, 0.72) for females. For the study period, the PR was 2.935 (95% CI: 2.478, 3.477; *p* < 0.001) in males compared to females. The prevalence was consistently higher for males than females in all of the study years. However, there was a greater overall percentage increase in prevalence from 2017 to 2021 for females compared to males (58% versus 26%).

In 2021, for hypnotics/sedatives, the prevalence was 1.42 (95%CI: 1.30, 1.55) for males and 1.13 (95%CI: 1.02, 1.24) for females. For the study period, the PR was 1.259 (95% CI: 1.187, 1.336; *p* < 0.001) in males compared to females. The prevalence was consistently higher for males than females in all of the study years and there was a greater overall percentage increase in prevalence from 2017 to 2021 for males compared to females (16% versus 9%).

**Table 4:** Trends in prevalence by psychotropic drug class and gender (5–15 years), overall % increase (2017–2021) and prevalence ratio (PR)

Drug class	Year					% increase 2017–2021	PR^b^ (M v F)
	2017	2018	2019	2020	2021		
ADHD medication	Prevalence^a^						
Male	5.74 (95%CI: 5.50, 6.00)	6.17 (95%CI: 5.91, 6,43)	6.57 (95%CI: 6.31, 6.84)	6.76 (95%CI: 6.49, 7.03)	7.31 (95%CI: 7.03, 7.59)	27%	4.497 (95% CI: 4.241, 4.769; *p* < 0.001)
Female	1.24 (95%CI: 1.13, 1.36)	1.34 (95%CI: 1.22, 1.46)	1.46 (95%CI: 1.34, 1.59)	1.54 (95%CI: 1.41, 1.67)	1.68 (95%CI: 1.55, 1.81)	35%	
**Antidepressants**							
Male	1.12 (95%CI: 1.01, 1.23)	1.33 (95%CI: 1.21, 1.45)	1.49 (95%CI: 1.37, 1.62)	1.68 (95%CI: 1.55, 1.82)	1.65 (95%CI: 1.52, 1.79)	47%	1.276 (95% CI: 1.063, 1.531; *p* = 0.009)
Female	1.04 (95%CI: 0.94, 1.15)	1.23 (95%CI: 1.12, 1.35)	1.37 (95%CI: 1.25, 1.49)	1.60 (95%CI: 1.47, 1.73)	1.84 (95%CI: 1.70, 1.98)	77%	
**Antipsychotics**							
Male	1.13 (95%CI: 1.02, 1.24)	1.24 (95%CI: 1.13, 1.36)	1.40 (95%CI: 1.28, 1.53)	1.47 (95%CI: 1.34, 1.60)	1.42 (95%CI: 1.30, 1.55)	26%	2.935 (95% CI: 2.478, 3.477; *p* < 0.001
Female	0.40 (95%CI: 0.34, 0.47)	0.42 (95%CI: 0.36, 0.49)	0.50 (95%CI: 0.43, 0.57)	0.57 (95%CI: 0.50, 0.66)	0.63 (95%CI: 0.55, 0.72)	58%	
**Hypnotics / sedatives**							
Male	1.22 (95%CI: 1.11, 1.34)	1.34 (95%CI: 1.23, 1.47)	1.36 (95%CI: 1.24, 1.48)	1.44 (95%CI: 1.32, 1.58)	1.42 (95%CI: 1.30, 1.55)	16%	1.259 (95% CI: 1.187, 1.336; *p* < 0.001
Female	1.04 (95%CI: 0.98, 1.20)	1.08 (95%CI: 0.98, 1.19)	1.05 (95%CI: 0.95, 1.16)	1.09 (95%CI: 0.98, 1.20)	1.13 (95%CI: 1.02, 1.24)	9%	

^a^Prevalence = as defined in Table 1

^b^PR = as defined in Table 1

### Seasonal differences in psychotropic medication use

**Fig. 3:**
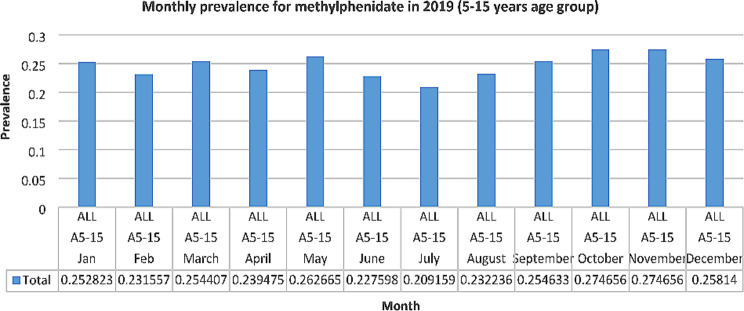
Monthly prevalence for methylphenidate in 2019

Monthly prevalence for the ADHD medication methylphenidate was examined for the 5–15 years group in 2019, to ascertain if there was any seasonal fluctuation in prevalence. It was decided to examine monthly prescribing for 2019 as this was before the COVID-19 pandemic began. In 2019, prevalence was lowest during the months of June, July and August and highest in October and November (See Fig. 3).

**Fig. 4:**
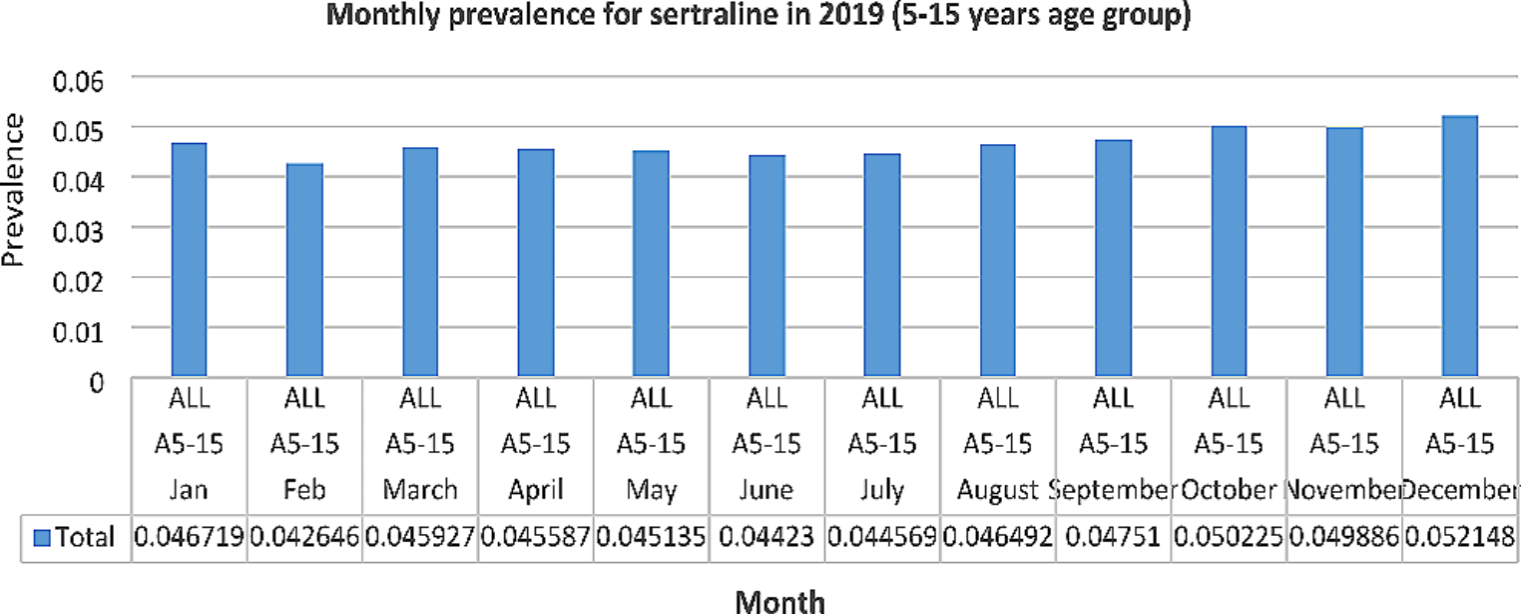
Monthly prevalence for sertraline in 2019

Monthly prescribing for the antidepressant sertraline was also examined for seasonal fluctuation for the 5–15 years group in 2019. Prevalence was lowest in February, June and July but then increased steadily up to December to a higher rate than at the beginning of 2019 (See Fig. 4).

**Fig. 5:**
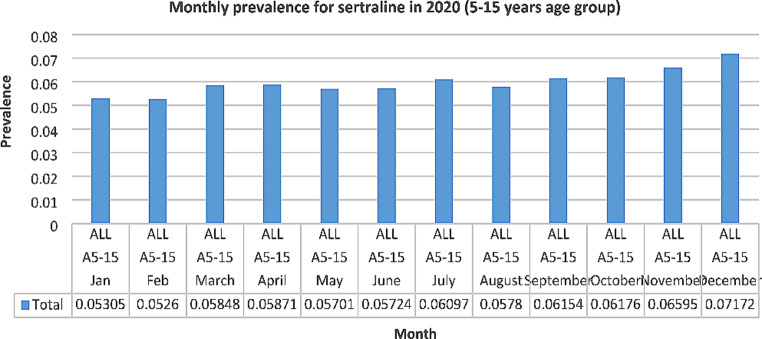
Monthly prevalence for sertraline in 2020

Monthly prescribing for sertraline was also examined in 2020 to determine if the start of the COVID-19 pandemic had any impact on prevalence. Similar to the pattern in 2019, prevalence decreased slightly in May, June and August but then increased steadily up to December to a rate higher than before the COVID-19 pandemic (36% increase from January 2020) (See Fig. 5).

### Comparison studies

In the past two decades, previous studies on psychotropic medication use in children/adolescents in Ireland have been undertaken, with increasing trends observed (Table 5). It is important to acknowledge that these studies were based on prescriptions dispensed to GMS patients only (which over-represents lower socio-economic groups) and not to the entire eligible population (as for our study). These studies also considered any dispensing of a medication in a calendar year to be of equal weight as dispensing of medication for all 12 months of a given year. Hence, given the different populations involved (i.e. lower socio-economic cohort only) and definitions of prevalence used, medication prevalences calculated in previous Irish studies are higher. Therefore, while we can observe trends reported by previous studies, we cannot compare prevalences with those from our study, as the comparison is not equivalent (refer to footnotes in Table 5 detailing prevalence definitions).

Recent international studies have also examined trends in psychotropic medication use, including studies from the US, Australia, the UK, the Netherlands and Scandinavia (Table 6). In these studies, as for our study, prevalences have been calculated per 1000 entire eligible population i.e. including all socioeconomic cohorts.

However, as for the previous Irish studies in Table 5, prevalences typically include any dispensing to a patient of a medication in a given year and are not calculated as averages of 12 monthly prevalences in a given year (as has been done for our study).

**Table 5:** Psychotropic medication prevalence data from previous Irish studies

Author(s)	Publishing year	Years studied	Study population (years)	Medicines	Most recent year prevalence reported	Prevalence* (per 1000 eligible population)
Boland et al.	2015	2002–2011	0–15	**ADHD medication (psychostimulants)**	2011	8.63
O’Sullivan et al.	2015	2002–2011	0–15	**Antidepressants**	2011	2.86
O’Sullivan et al.	2015	2002–2011	0–15	**Benzodiazepines**	2011	5.33
MacAvin et al.	2020	2005–2015	0–24	**ADHD medication**	2015	8.36
Conlan et al.	2021	2005–2015	0–24	**Antipsychotics**	2015	3.97 (< 16 years)

*Prevalences are based on patients receiving at least one prescription per 1000 GMS eligible population

**Table 6:** Psychotropic medication prevalence data from recent international studies

Author(s)	Publishing year	Years studied	Geography	Study population (years)	Medicines	Most recent year prevalence reported	Prevalence (per 1000 eligible population)*
Candon et al.	2021	2014–2018	US	≤ 21	**Antipsychotics**	2018	8.0
Cao T.X.D. et al.	2021	2000–2018	UK	5–17	**Antidepressants**	2018	5.91
Bais et al.	2022	2010–2019	Netherlands	≤ 19	**Antipsychotics**	2019	7.9 (7–12 years)
Klau et al.	2022	2011–2018	Australia	0–18	**All psychotropic medication**	2018	36.2 (≤ 19 years) 16.6 (5–9 years) 54.1 (10–14 years)
Sørensen et al.	2022	2010–2020	Scandinavia	5–19	**ADHD medication**	2020	35 (Sweden) 22 (Denmark, Norway)
Bliddal et al.	2023	2017–2022	Denmark	5–24	**All psychotropic medication**	2022	8.91 (5–11 years) 22.59 (12–17 years)
Radojčić et al.	2023	2000–2019	England	3–18	**Antipsychotics**	2019	1.05

*Prevalences have been calculated per 1000 entire eligible population, including any dispensing of a medication in a given year

## Discussion

### Statement of principal findings

The findings from this study suggest an increasing trend in prevalence from 2017 to 2021 for all selected psychotropic medications in children/adolescents aged 5–15 years, as well as for the stratified age groups, 5–11 years and 12–15 years. Increasing prevalence trends were also observed for the individual selected drug classes. Between the four main drug classes, the largest increase in prevalence in children/adolescents aged 5–15 years was observed for antidepressants, followed by antipsychotics, ADHD medication and hypnotics/sedatives.

### Trends in psychotropic medication use

In the past two decades, previous studies on psychotropic medication use in children/adolescents in Ireland have been undertaken, with increasing trends observed. For example, the rate of psychostimulant use for ADHD in Ireland (0–15 years, GMS patients only, thus over representing lower socio-economic groups) increased significantly between 2002 and 2011 (3.77 vs. 8.63/1000 GMS population) [10]. Between 2005 and 2015, another study found that there was an increase in the use of ADHD medication in patients less than 25 years (5.61 vs. 8.36/1000 GMS population), along with a significant increase in the percentage taking concomitant antidepressants (2%vs 6%) [11]. A separate Irish study on the use of antidepressants in children between 2002 and 2011, reported a decrease between 2002 and 2008 (coinciding with the Irish Medicines Board (IMB) warning against the treatment of childhood depression with selective serotonin reuptake inhibitors (SSRIs) due to increased risk of suicide), but a subsequent increase between 2008 and 2011 (2.61 vs. 2.86/1000 GMS population). Between 2002 and 2011, while benzodiazepine use in children 0–15 years decreased (8.56 vs. 5.33/1000 GMS population), a significant proportion of children (GMS patients) received long term prescriptions for benzodiazepines [12], but approximately 25% of these were co-prescribed an antiepileptic, suggesting treatment for medical indications other than psychiatric indications. GMS dispensing rates for antipsychotics in Irish children/adolescents 0–16 years were stable across the decade 2005–2015 (3.94 vs. 3.97/1000 GMS population) [13].

Following on from past Irish research, our study also suggests an increasing trend in psychotropic medication use in children/adolescents. Of note, because rates were found to be lower than international comparators in previous Irish studies, this increasing trend is starting from a low base rate. The increasing trend of psychotropic medication use is likely due to greater awareness of mental health conditions, coupled with increased awareness of treatment guidelines recognising the evidence base for medication use.

Similar to what has been observed in previous Irish studies, international literature indicates that the prescribing prevalence of psychotropic medications in children/adolescents in other countries such as the US, Australia, New Zealand, the UK, the Netherlands, Denmark, Sweden and Norway is still higher than in Ireland [2, 3, 14–22]. For example, according to recent international literature (Table 6), the prevalence of psychotropics was shown to be higher than Ireland in an Australian study from 2018 (5–9 years, prevalence = 16.6/1000 population; 10–14 years, prevalence = 54.1/1000 population) [3] and in a Danish study (5–11 years, prevalence = 8.91/1000 population; 12–17 years, prevalence = 22.59/1000 population) [21]. In Sweden, Denmark and Norway, prevalences for ADHD medication in 2020 (5–19 year olds) were 35, 22 and 22/1000 population respectively [18]. In the UK, 5.9/1000 population of 5–17 year olds were taking antidepressants in 2018 [15]. In the Netherlands, 7.9/1000 population of 7–12 years olds were taking antipsychotics in 2019 [16]. Similarly, in the US, 8/1000 population of children/adolescents less than 19 years were taking antipsychotics [22]. By comparison, our study estimates that in 2021, 8.46/1000 population of Irish children/adolescents 5–15 years were dispensed a psychotropic medication (from the medications selected for the study), which includes 4.43/1000 population taking medication for ADHD, 1.75/1000 population taking antidepressants, and 1.01/1000 population taking antipsychotics. Of note, in England, 1.05/1000 population of 3–18 year olds were taking antipsychotics in 2019, which is similar to the rate in Ireland [23].

Literature has shown that there has been an increase in psychotropic medication use in children/adolescents globally [4] and the question of whether this increase is justified has been much debated. It has been suggested by some studies that children/adolescents with mental illness are undertreated and that an increase in prescribing is justified, particularly in conjunction with data on prevalence rates of mental health problems [24]. In particular, some studies have reported suboptimal levels of diagnosis and treatment of chronic mental disorders such as ADHD [25].

In our study, for ADHD medication, the prevalence observed for the 5–15 years group in 2021 (0.44%) is low when compared to the estimated prevalence of ADHD in Ireland for similar age groups (3–7%) and international literature [26–28]. Further research is needed to explore this comparatively low prevalence of ADHD medication in Irish children/adolescents. There may be many reasons why medication initiation and adherence/persistence in ADHD treatment can be suboptimal. Possible reasons include socioeconomic factors such as stigma, poverty and low education levels; patient-related factors such as increased age and parent/family influence; drug-related factors such as side-effects and perceived lack of efficacy and therapy-related factors such as inconvenient dosing regimens and medication costs [29, 30]. Reluctance to initiate ADHD treatment and poor adherence/persistence with treatment is a recognised challenge; families with children/adolescents with ADHD should be supported using appropriate interventions [31, 32].

In the case of other mental disorders such as depression, literature has suggested overdiagnosis in children/adolescents, along with overprescribing of psychotropic medication [33]. Recently in Ireland, CAMHS has been under scrutiny and there have been concerns of overprescribing of psychotropic medications. However, comparison of our study results with international data has shown that these concerns are not supported by Irish reimbursement data, as rates of psychotropic use in children/adolescents in other countries are generally higher than in Ireland. This is corroborated further by the National Audit of Prescribing in Child and Adolescent Mental Health Service (July 2023) [34], which did not find evidence of overprescribing in Ireland (less than 50% of children attending CAMHS were prescribed medication) and found that most patients (95%) had been prescribed medication by a consultant or in consultation with a consultant. The audit covered all CAMHS teams nationwide and was undertaken as a consequence of the Maskey Report (January 2022), which highlighted that CAMHS care in South Kerry, Ireland (a regional CAMHS Ireland centre) was sub-optimal in terms of care planning, diagnostic accuracy and medication prescribing.

With regards to higher levels of psychotropic medication use in children/adolescents internationally as compared to Ireland, further research is required to determine the reasoning behind this. It has been acknowledged in the literature that use of pharmacotherapy for children/adolescents with mental disorders varies widely across countries and this variability may reflect differences in diagnostic systems, clinical practice guidelines, drug regulation, health services organisation, availability and allocation of financial resources, and cultural attitudes towards childhood behavioural and emotional disturbances [35]. Additionally, individual clinicians’ practice, training and confidence are all known to be associated with different rates of prescribing and different rates of patient nonadherence [36]. It has been suggested that cultural context may exert a greater influence on the management of psychiatric disorders than on other areas of medicine. For example, in some cultures e.g. Arab culture, there may be increased levels of reluctance, stigma or fear surrounding the use of psychotropic medication, although such stigma is nuanced and can vary by country [37, 38]. By contrast, in the US, guidelines for conditions like ADHD tend to advocate liberally for medication use; in Europe, medication is restricted to more severe presentations [20].

Further research is warranted to determine if the observed variability in medication prescription and use leads to different clinical outcomes, with respect to either benefit or adverse effects [35]. For example, do higher levels of ADHD prescribing in the US or Scandinavia translate into a better short and longer term prognosis as compared with countries with lower prescribing levels, such as Ireland?

### Gender differences in psychotropic medication use

In the 5–15 years age group, prevalence for all selected psychotropic medications was found to be two to three times higher for males than females for all study years, with a greater increase in prevalence over the study period for females (41%) than males (28%). Differences in prevalence between genders were also found for the individual drug classes. For ADHD medication, prevalence in 2021 was higher for males than females. This may align with the increased prevalence in ADHD among males as compared to females. However, this male predominance is also found when examining rates of antipsychotic and hypnotic/sedative drug classes. The pattern of increased prescribing in males compared to females within ADHD has been reported in prior research (Irish and international) [11, 18, 39].

For antipsychotics in 2021, prevalence for males was approximately twice that for females. Similar ratios have been reported in international studies [3, 16, 23], but not in New Zealand, where antipsychotic prescription rates were similar among boys and girls [14].

Further research is recommended to fully explore the implications of gender differences, along with country differences on both baseline and increasing prescribing rates.

For antidepressants, prevalence was consistently higher for males than females from 2017 to 2020 but in 2021, this gender ratio reversed, with rates of antidepressant use higher among females. Previous Irish and international studies highlight that the prevalence for antidepressants in girls increases exponentially post menarche [15, 40, 41]. Further research is necessary to explore this change, and to what extent the COVID-19 pandemic may have played a part, including stratification by age as well as gender.

### Seasonal differences in psychotropic medication use

In 2019, for the ADHD medication methylphenidate, prevalence was lowest during the months of June, July and August and highest in October and November. This suggests an understandable degree of seasonal fluctuation, with decreased use during the summer months and increased use in October and November when children/adolescents have returned to school. Seasonal fluctuation for ADHD medication has also been reported in the literature [42] but further analysis is recommended to ascertain if the fluctuation suggested by the results of our study is statistically significant. A broadly similar pattern was seen in 2019 for the antidepressant sertraline, in that prevalence was lowest in February, June and July and then increased steadily up to December to a rate higher than at the beginning of the year. This fluctuation may reflect heightened anxiety experienced upon return to school in September and during school term-time but further research is needed to explore this fully. It is acknowledged that lower prevalences in February may be due to this month being the shortest of the year. However, the community drugs reimbursement schemes in Ireland require a claim each calendar month; Hence, the effect of the shorter month should be small.

### Effects of the COVID-19 pandemic on trends in psychotropic medication use

The results of our study suggest that the use of psychotropic medication in children/adolescents has increased in Ireland from 2017 to 2021. However, it is difficult to determine if this increase relates to the COVID-19 pandemic. Monthly dispensing for sertraline was examined for the 5–15 years age group in 2020 to look for fluctuations in use and to determine if the beginning of the COVID-19 pandemic had any impact on supply / access to medication. Similar to the pattern in 2019, prevalence decreased slightly in May, June and August, then increased steadily up to December to a rate higher than before the COVID-19 pandemic (36% increase from January 2020). As with the monthly pattern for sertraline in 2019, this may relate to the start of the school year in September and resulting anxiety experienced by some children/adolescents, but further research should be undertaken to explore this.

It has been acknowledged in the literature that the pandemic has had a severe impact on mental health and wellbeing globally [43]. For example, the results of a Danish study suggest that children/adolescents experienced an increase in rates of psychiatric disorder diagnoses and psychotropic treatment during the pandemic, which was most pronounced among those aged 12 to 17 years [21]. Further research and an extended study of Irish trends in psychotropic medication use may be required to determine the short- and long-term effects of COVID-19 on the mental health of children/adolescents [44].

## Limitations

This study has a number of strengths including the large study size and accurate ascertain of medicines. However, there are also limitations associated with the study. Firstly, although the study included data for the four main classes of psychotropics, some medications were not included e.g. clonidine (not included due to its multiple indications and the dataset would not allow selection of psychiatry indications) and lithium. Secondly, we did not calculate the proportion of individuals receiving psychotropic medication at any time in a given calendar year. Instead, we calculated and defined yearly prevalence as the average number of patients in receipt of each medication per month per 1000 eligible population. As discontinuation rates of psychotropic medications in youth are high, it was felt that an average number of patients dispensed medication per month averaged over a year would better reflect actual medication use compared to a prevalence of those who received any prescription supply during that year. Thirdly, the literature we have used for comparison, including previous Irish and international studies and more recent international comparators, has used different databases, methods of measuring prevalence and licensing and reimbursement policies for medications. There is high heterogeneity across the various studies, in terms of age groups, cohort size and year in which the data are assessed. Fourthly, our study might be underestimating prevalence as it excludes data for private patients, although this data should be low as all patients in the 5–15 years group are eligible to receive the medications selected for the study under the HSE-PCRS-LTI scheme without payment. Fifthly, for some medications, there may be overlap because some medications are prescribed for indications other than psychotropic indications e.g. use of midazolam in epilepsy. Additionally, patients taking more than one medication have been counted twice in our analysis, although this would suggest that actual rates are lower.

No data was available on the professional details of clinicians initiating prescriptions i.e. specialist versus generalist, or indications for prescriptions. It would be beneficial in future research studies if linkage between prescribing and clinical data, such as diagnosis and outcomes, were possible. This would enable research questions on the appropriateness of medication and the likely impact on short-, medium- and long-term outcomes, particularly in the context of lower use of psychotropic medications in Ireland as compared to other countries. Finally, another limitation may be that psychotropic drugs can be simultaneously over- and under-prescribed in a given population, to the extent that some patients receive medication that is not truly indicated for them, while other patients may not receive medication which is indicated. This is difficult to discern from population-level data.

## Conclusion

There has been an increase in psychotropic medication use in children/adolescents in Ireland between 2017 and 2021, suggesting that the upward trajectory witnessed in 2005–2015 has continued. Despite increasing trends, however, comparison with the literature shows that prevalences of psychotropic medications in Ireland remain lower than international comparators. Across all similar geographies, year on year increases in psychotropic medication use in children/adolescents were common, but our results suggest that the increase observed for Ireland still failed to outstrip other jurisdictions. Despite the increasing trend, Ireland is operating at a lower rate base level than similar countries. As it is unlikely that this reflects lower incidences of mental illness in Ireland, or increased access and availability of non-pharmacological evidence-based treatments, our study has shown that public concerns of systematic overuse of psychotropic medication in Irish children/adolescents are not supported by reimbursement data. Our study recommends that further research is undertaken to explore the reasons behind and clinical impact of lower rates of psychotropic medication use in children/adolescents in Ireland compared to other countries and the implications of gender differences in psychotropic medication use. Furthermore, future studies should examine the significance and merits of observed seasonal fluctuations in psychotropic medication use and the short- and long-term effects of COVID-19 on the mental health of children/adolescents.

## Electronic supplementary material

Below is the link to the electronic supplementary material.

Supplementary Material 1
