# Supplementary material for: Recent trends in psychotropic medication use in children and adolescents in Ireland
Source: Eur Child Adolesc Psychiatry. 2024 Aug 8;34(3):997–1009. doi: 10.1007/s00787-024-02530-7 (PMC11909023; doi:10.1007/s00787-024-02530-7)
Supplement: Supplementary file 1 — Supplementary Material 1 [file 787_2024_2530_MOESM1_ESM.docx]

**Supplementary material**

**Ref: Submission ID 1afd712b-8c72-4ef1-9fa2-78b1fcbdfd5b**

**Appendix 1: List of psychotropic medications for which prevalence data was extracted**

| **Class of psychotropic medication** | **Medication** |
| --- | --- |
| **ADHD medication** | Atomoxetine |
|  | Guanfacine |
|  | Lisdexamphetamine |
|  | Methylphenidate |
|  | Modafinil |
| **Antidepressants** | Agomelatine |
|  | Amitriptyline |
|  | Bupropion |
|  | Citalopram |
|  | Clomipramine |
|  | Dosulepin |
|  | Doxepin |
|  | Duloxetine |
|  | Escitalopram |
|  | Fluoxetine |
|  | Fluvoxamine |
|  | Imipramine |
|  | Lofepramine |
|  | Mirtazapine |
|  | Moclobemide |
|  | Nortriptyline |
|  | Paroxetine |
|  | Phenelzine |
|  | Reboxetine |
|  | Sertraline |
|  | Tranylcypromine |
|  | Trazodone |
|  | Trimipramine |
|  | Tryptophan |
|  | Venlafaxine |
| **Antipsychotics** | Amisulpride |
|  | Aripiprazole |
|  | Asenapine |
|  | Chlorpromazine |
|  | Flupentixol |
|  | Fluphenazine |
|  | Haloperidol |
|  | Levomepromazine |
|  | Lithium |
|  | Olanzapine |
|  | Paliperidone |
|  | Perphenazine |
|  | Pimozide |
|  | Prochlorperazine |
|  | Quetiapine |
|  | Risperidone |
|  | Sulpiride |
|  | Trifluoperazine |
|  | Ziprasidone |
|  | Zuclopenthixol |
| **Hypnotics / sedatives** | Alprazolam |
|  | Bromazepam |
|  | Chlordiazepoxide |
|  | Clobazam |
|  | Diazepam |
|  | Flurazepam |
|  | Lorazepam |
|  | Lormetazepam |
|  | Midazolam |
|  | Nitrazepam |
|  | Potassium chlorazepate |
|  | Prazepam |
|  | Temazepam |
|  | Triazolam |
|  | Zolpidem |
|  | Zopiclone |
